# Efficacy and Safety of Combined Surgeries including Intraocular Direct Cyclophotocoagulation with a 532 nm Laser to Treat Advanced Neovascular Glaucoma

**DOI:** 10.1155/2021/9938928

**Published:** 2021-07-07

**Authors:** Xiaomin Chen, Tian Zheng, Wen Zeng, Xia Fu, Shun Wang, Weijuan Zeng, Min Ke

**Affiliations:** Department of Ophthalmology, Zhongnan Hospital, Wuhan University, Wuhan, China

## Abstract

**Purpose:**

The purpose of this study was to assess the efficacy and safety of intraocular direct cyclophotocoagulation (IDCP) using a 532 nm laser with combination treatment in reducing intraocular pressure (IOP) for patients with advanced neovascular glaucoma (NVG) with angle closure. In addition, we sought to determine the success rate and frequency of complications and explore an optimized solution to reduce the number of topical medications and the pain of patients.

**Methods:**

A retrospective case note review of all patients undergoing combined treatment including IVR, phaco, PPV, PRP, and IDCP from January 2017 to June 2018.

**Results:**

In total, 24 consecutive patients (25 eyes) were reviewed. The mean IOP was significantly decreased from 42.2 ± 8.5 mmHg preoperatively to 15.56 ± 2.0 mmHg (*P* < 0.0001), and no neovascularization of the iris (NVI) reoccurred at month 18. The number of medications used was reduced from 2.72 ± 0.45 preoperatively to 0.87 ± 0.40 at month 18 (*P* < 0.0001).

**Conclusions:**

During 18 months of follow-up, the combined treatments were safe and had a significant IOP-lowering effect. This study provides a new method of performing IDCP with a 532 nm laser, allowing for the use of internal cyclophotocoagulation without extra equipment.

## 1. Introduction

Neovascular glaucoma (NVG) is a serious and potentially blinding secondary glaucoma, often resulting from ocular ischemia caused by proliferative diabetic retinopathy (PDR), retinal vein occlusion (RVO), or other ischemic retinal disorders [1, 2]. In advanced NVG, the neovascularization invades the anterior chamber angle and forms a fibrovascular membrane [3, 4], resulting in the formation of peripheral anterior synechiae (PAS) and exceeding the Schwalbe line. At that stage, the elevated IOP is hard to control and eventually leads to irreversible and massive vision loss and severe pain [2]. Patients in developed countries often do not progress to this late stage because they can access treatment in an early stage, but it is not uncommon for patients in China to develop to this stage. If the condition remains untreated, enucleation is necessary to relieve the pain.

Previous studies have reported that anti-VEGF agents for NVG cause a rapid and substantial regression of neovascularization of the iris (NVI) and the anterior chamber angle (NVA) [5, 6]. However, due to the short half-life of these agents, this regression is transient [5, 7]. Anti-VEGF treatments cannot treat the NVG itself, only serving as adjunct therapy for antiglaucoma surgery [8]. Currently, panretinal photocoagulation (PRP) is the standard treatment [2, 9, 10]. However, this is unsuitable for patients with media opacity such as cataract or vitreous hemorrhage. Furthermore, elevated IOP is refractory to both medical management and surgical intervention in most cases of advanced NVG with angle closure [11].

Since Uram [12] first applied endoscopic cyclophotocoagulation (ECP) to patients with refractory open angle glaucoma in 1995, studies of ECP have been increasingly reported in cases of refractory glaucoma, including NVG [13–15]. Considering the complications related to trabeculectomy, such as infection, hypotony, and leakage or failure of the filtering bleb, ECP has been gradually accepted in the United States [16–19]. In contrast, due to the requirement of an expensive device, ECP is less common in Asia [20].

In our study, we did not require dedicated ECP equipment to observe the ciliary body but exposed it by appropriate pressure; in addition, we used 532 nm laser instead of 810 nm laser.

This study aimed to assess retrospective midterm results of the efficacy and safety of intraocular direct cyclophotocoagulation (IDCP) with 532 nm green laser combined with pars plana vitrectomy (PPV), phacoemulsification cataract extraction (phaco), PRP, and intravitreal ranibizumab (IVR) in cases of NVG.

## 2. Methods

### 2.1. Patients and Inclusion Criteria

This retrospective study was approved by the Institutional Review Board of the Zhongnan Hospital of Wuhan University (2019052) and performed in accordance with the principles of the Declaration of Helsinki. This study reviewed the medical records of 25 eyes from 24 advanced NVG patients with angle closure treated between January 2017 and June 2018. Confidentiality was maintained when the records were accessed.

NVG was defined as IOP > 21 mmHg with the presence of NVI or NVA. Patients were included in the study based on the following criteria: (i) diagnosis of NVG; (ii) angle closure of 180° or more with PAS formation; and (iii) IOP > 30 mmHg with the maximum number of antiglaucoma medications. Exclusion criteria included (i) active ocular infection; (ii) any contraindication of intraocular injection or surgery, such as high risk of bleeding, pregnancy, or infection; (iii) corneal decompensation or opacity-preventing examination or surgery after IOP was normalized in response to anterior chamber paracentesis; and (iv) lens and vitreous body sufficiently transparent to allow PRP with a slit lamp.

### 2.2. Surgical Techniques and Procedure

All subjects were treated with IVR initially followed by PPV, phaco, PRP, and IDCP (532 nm laser) within 5 days. Ranibizumab (Novartis, Switzerland) injection was performed in an operation room. After aseptic preparation (5% povidone-iodine solution) and application of topical anesthetic eye drops (proparacaine hydrochloride, 0.5%; Alcaine, Alcon, USA), ranibizumab solution (10 mg/mL, 0.05 mL) was injected using a 27-gauge needle.

**Figure 1:**
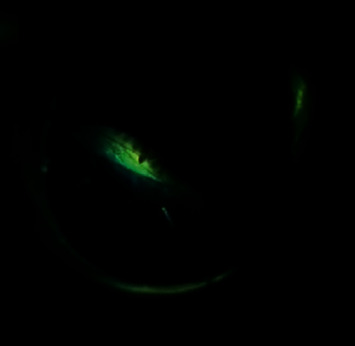
The ciliary processes are clearly visible in the visual field of the operation microscope when performing IDCP.

Removal of the lens combined with vitrectomy made visualizing the ciliary processes possible. PRP (laser settings: continuous mode, energy 80–140 mW, and exposure time 400 ms) and IDCP (laser settings: continuous mode, energy 220–300 mW, and exposure time 400 ms) were performed using a 532 nm green laser (OcuLight GL; Iridex, USA) after PPV and phaco. The scleral depression was used to bring the ciliary processes under direct visualization, and then IDCP was applied. With proper pressure, the ciliary processes were fully exposed to the visual field of the microscope (Figure 1). A total of 180°–270° of ciliary processes were treated, with whitening and shrinkage of the ciliary processes as the treatment end point.

### 2.3. Outcome Measurement and Follow-Up

All subjects underwent ophthalmologic examinations including detailed medical histories, best-corrected visual acuity (BCVA), anterior segment and fundus examinations, IOP measurements by iCare (TA01i; Tlolat Oy, Finland), and anterior chamber angle examinations with optic coherence tomography (OCT; Zeiss CIRRUS HD OCT 5000, Germany). BCVA was recorded using logarithm of the minimal angle of resolution (LogMAR) format (counting fingers, 2.0; hand movements, 3.0; light perception, 4.0; no light perception, 5.0).

The medical records included age, sex, BCVA, pre-existing ischemic ocular disorders, number of prescribed topical antiglaucoma medications, and prior treatments for original ischemic ocular disorders. The NVI grade was assessed by the Weiss and Gold classification with 4 stages of neovascularization, based on the area of new vessels in the iris: grade 1, surface neovascularization of the pupillary zone of the iris, involving ≤ 2 quadrants; grade 2, surface neovascularization of the pupillary zone of the iris, involving > 2 quadrants; grade 3, in addition to the pupillary zone, neovascularization of the ciliary zone of the iris and/or ectropion uveae involving 1 to 3 quadrants; and grade 4, neovascularization of the ciliary zone of the iris and/or ectropion uveae involving ≥ 3 quadrants [21]. Grading of NVI was performed by a single glaucoma specialist. The degree of anterior chamber angle closure and the location of PAS were analyzed in the OCT image.

The efficacy of the combined surgery was classified into one of three categories: “Complete success” was defined as IOP of ≥ 6 mmHg and ≤ 21 mmHg without any use of antiglaucoma medications or further glaucoma surgery. “Qualified success” was defined as IOP < 21 mmHg with the use of topical antiglaucoma medication. “Surgical failure” was defined as IOP >21 mmHg even with antiglaucoma medication and the necessity for additional surgical treatment to control IOP.

Pain was assessed and recorded by numerical rating scale (NRS), ranging from 0 (no pain) to 10 (worst imaginable pain). Surgical complications and postoperative antiglaucoma medications were also reviewed. The patients were followed for at least 18 months after the operation. The staff members performing the NRS, BCVA, and IOP assessment were not involved in implementing the surgeries.

### 2.4. Evaluation of Acute Structural and Histopathological Changes in Human Donor Eyes after IDCP (532 nm Laser)

Two human donor eyes were obtained from the Red Cross Eye Bank of Wuhan, Tongji Hospital, China. Both eyes with brown iris were harvested from Chinese individuals without eye disease history within 4 hours after death. The anterior segment was mounted with the lens facing up and the cornea down. The ciliary processes were clearly visible under a microscope. IDCP was performed using the 23-gauge laser probe with a 532 nm green laser, which directly faced the ciliary processes. The laser settings were the same as those used in patients: continuous mode, energy 220–300 mW, and exposure time 400 ms. A total of 180° of ciliary processes was treated, with whitening and shrinkage of the ciliary processes as the end point. The treatment was initiated at the 12 o'clock position, which was identified by the Vicryl suture and preceded clockwise. The remaining 180° were untreated as the normal control. Sections were stained with hematoxylin and eosin/phloxine and examined histologically using standard light microscopy. This procedure followed a published protocol [22].

### 2.5. Statistical Analysis

Pretreatment baseline IOP, BCVA, NVI, and numbers of medications were compared with posttreatment follow-up examination values using nonparametric Wilcoxon signed-rank paired-tests. Results are presented as mean ± standard deviation (SD). A *P*-value of <0.05 was considered statistically significant.

## 3. Results

### 3.1. Baseline Characteristics

The demographics and baseline characteristics of included patients are shown in Table 1. A total of 24 patients (25 eyes), who received the combined treatment and were followed up for 18 months, were included in this study. Among them, 11 were male and 13 were female. The mean age was 58.3 ± 8.1 years, and IOP was 42.2 ± 8.5 mmHg. Patients received an average of 2.72 ± 0.45 IOP-lowering medications before treatment. The underlying ophthalmic conditions causing NVG were PDR (16 eyes, 64%), central retinal vein occlusion (6 eyes, 24%), and ocular ischemic syndrome (2 eyes, 8%).

### 3.2. IOP

**Figure 2:**
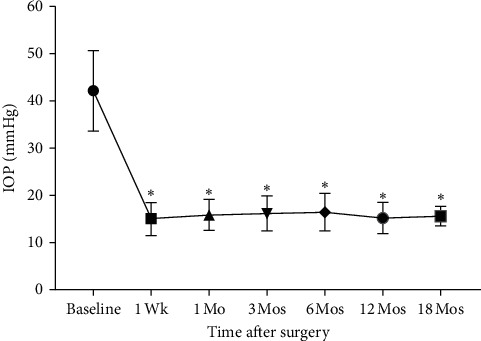
Change in IOP throughout the study period. Compared with baseline, the IOP (mean ± SD) was significantly decreased at all time points after the combined surgery (^*∗*^*P* < 0.0001).

Normalized IOP (≤ 21 mmHg) with rapid neovascular regression was achieved in all 25 eyes within 1 week of the procedure. At every time point after the combined surgery, the IOP was significantly decreased compared with baseline (*P* < 0.0001). The postoperative change in IOP is shown in Figure 2.

### 3.3. NVI and the Degree of Anterior Chamber Angle Closure

**Figure 3:**
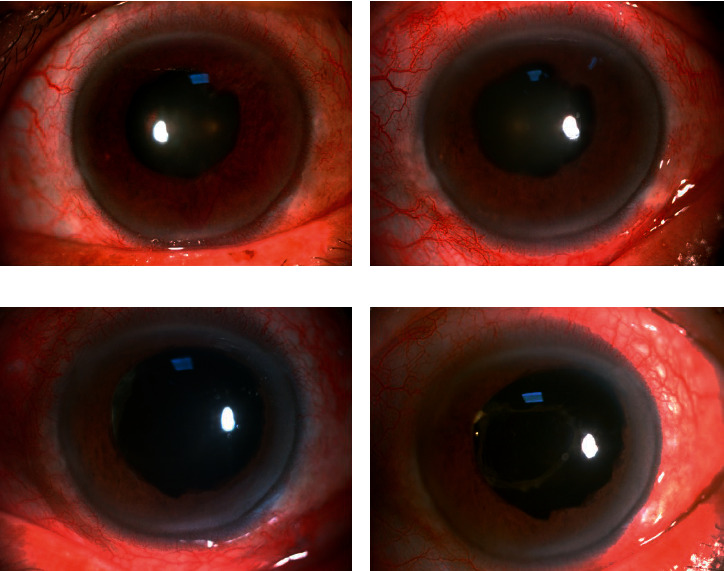
Anterior segment photography of an NVG patient before and after the combined treatment. (a) Massive neovessels and ectropion uveae were observed on the iris and the pupil prior to surgery. (b) The same eye 4 days after IVR, showing neovessel regression. (c, d) The same eye 10 days and 3 months after combined surgeries; no return of neovessels was observed.

**Figure 4:**
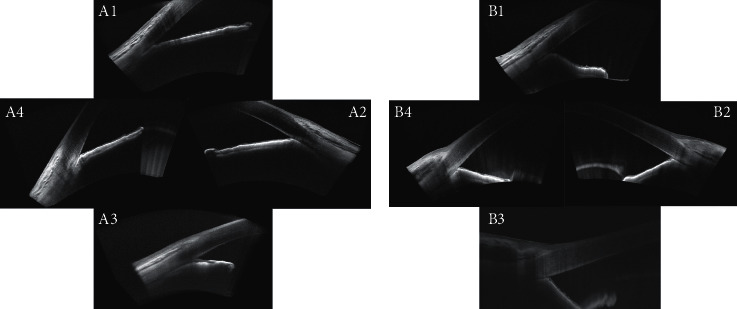
Anterior chamber angle OCT images of an NVG patient before and after the combined surgery. A1–A4, baseline images showing complete angle closure. B1–B4, the same eye, 10 months after surgery, showing that the angle remains closed. Images A1/B1, A2/B2, A3/B3, and A4/B4 represent 12, 3, 6, and 9 o'clock, respectively.

At baseline, 20 eyes (80%) were graded as exhibiting advanced NVI (grade 3 or 4), and all eyes had ≥ 180°of anterior chamber angle closure (Table 1). After IVR, NVI greatly regressed in all patients within 5 days and did not return within the 18-month follow-up period (Figure 3). However, the closed anterior chamber angle did not reopen (Figure 4).

### 3.4. BCVA and Pain Relief

The change in BCVA after the surgery is shown in Table 2 (*P*=0.0111). Compared with baseline, no eyes deteriorated, 3 (12%) remained stable, and 22 (88%) showed improved BCVA. Eye pain (NRS score) was significantly relieved from the first day after surgery (before surgery, 6.28 ± 1.59; first postoperative day, 1.68 ± 0.69, *P* < 0.0001) and nearly disappeared 1 month later (0.24 ± 0.44, *P* < 0.0001).

### 3.5. Success and Medications

Three months after the combined surgery, 22 eyes (88.0%) achieved complete success, defined as IOP between 6 and 21 mmHg without any use of antiglaucoma medications or further glaucoma surgery. After 6 months, 20 eyes (80.0%) retained that status, and 13 eyes (52%) at 18 months. Qualified success (target IOP achieved through topical antiglaucoma medication) was achieved in 3 eyes (12.0%) after 3 months and increased to 5 eyes (20.0%) at 6 months and 6 eyes (24%) after 18 months. No patients required additional antiglaucoma surgery within the first 12 months. Throughout the study period, 6 eyes (24%) received additional surgical intervention. Among the eyes exhibiting residual glaucoma after combined treatment, the IOP was not more than 28 and could be successfully controlled with 1 or 2 antiglaucoma medications. Patients with controlled elevated IOP would meet with much lower complications of filtering surgery because of former anti-VEGF and PRP treatment.

### 3.6. Complications

Postoperative complications are summarized in Table 3. Anterior segment inflammation was the most common complication, occurring in 14 eyes (56%), and was well controlled by topical atropine and dexamethasone. An additional 3 eyes (12%) exhibited hyphema, which was completely absorbed within 2 weeks after the combined surgery. There was no occurrence of postoperative pain, intraocular hemorrhage, choroidal detachment, or hypotony.

### 3.7. Comparison of Acute Structural and Histopathological Changes in Human Cadaver Eyes after IDCP

**Figure 5:**
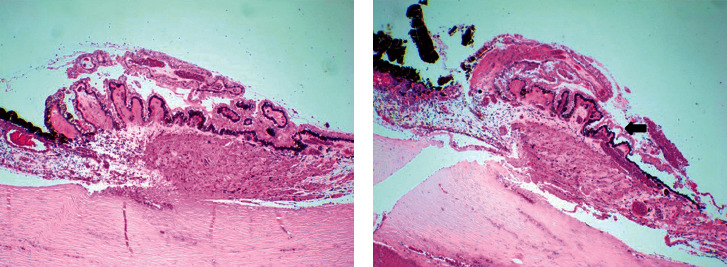
Light microscopy of ciliary processes (40x). (a) Normal ciliary processes showing normal-appearing stroma and the pigmented and nonpigmented ciliary epithelium. (b) IDCP-treated ciliary processes showing destruction of the nonpigmented epithelium and its detachment from the pigmented epithelium (straight arrows).

Compared with normal, untreated human eye tissue, IDCP-treated human eyes showed destruction of the nonpigmented epithelium and its detachment from the pigmented epithelium (Figure 5). The laser-treated areas showed no obvious destruction of the processes' stroma and ciliary muscle. The architectural damage did not extend into the ciliary muscle layer.

## 4. Discussion

In this study, we evaluated the efficacy and safety of combining IDCP with a 532 nm laser with standard treatment in patients with advanced NVG. This technique, if successful, would make intraocular direct cyclophotocoagulation available to patients in less-developed countries, where the cost of an endoscope is prohibitive. Our results showed a rapid disappearance of neovascularization and normalization of IOP, a reduction in the number of medications required, pain relief, and few postoperative complications for 18 months after the procedure.

Proper management for NVG includes the treatment of both the underlying disease and the elevated IOP [2, 23]. Although PRP along with intravitreal anti-VEGF antibody monotherapy is the standard treatment regimen, these cannot decrease the IOP in patients exhibiting advanced NVG with angle closure. Moreover, conventional surgical interventions to control IOP and prevent disease progression, including trabeculectomy, glaucoma drainage devices (GDD), and trans-scleral cyclophotocoagulation [1, 2, 23–29], have a high rate of failure and complications in these patients. PPV and phaco required to perform PRP and IDCP with 532 nm laser in our study because they allowed the use of external laser in patients with lens and vitreous opacity. PRP was used to treat the primary ischemic retinopathy of NVG, while IVR created a window period for surgery. The neovascularization would disappear for a short period after injection, allowing the operation to be carried out safely and efficiently. In this study, all patients were diagnosed with the advanced NVG in which the closure angle was over 180°. We used PPV, phaco, PRP, and IVR not to reduce IOP but to control the other symptoms of NVG, allowing us the opportunity to perform IDCP to control IOP.

While ECP has shown excellent results in patients with refractory glaucoma, including those with NVG, the technique rarely has been conducted in China, due to difficulties in accessing the necessary endoscope. In our study, the ciliary processes were photocoagulated with a 532 nm laser probe after PRP. This technique, IDCP, did not require an additional device. Both the 532 nm green laser and the 810 nm diode laser are thermal lasers; the main difference between them is that the diode laser penetrates deeper than the green laser. Decreased penetration, however, is an advantage when the intraocular laser probe is directed at the ciliary processes because the green laser is less likely to damage the deep matrix.

IDCP can be used to achieve the same effect as ECP, including observing the reaction of ciliary tissue to photocoagulation in real time and adjusting the parameters, as well as strictly controlling the degree of ciliary body damage to avoid insufficient or excessive destruction of ciliary processes. The low laser energy (220–300 mW) and brief exposure time (400 ms) used in this study resulted in only mild to moderate inflammation. Moreover, our results showed a rapid disappearance of neovascularization, normalization of IOP, a reduction in the number of medications required, pain relief, and few postoperative complications.

In our study, we have found that it is simple and convenient to obtain clear images of the anterior chamber angle using OCT, and that this technique is superior to gonioscopy in patients with NVG. The OCT images can be used to determine whether the angles are open or closed, as well as the position or quadrant of closure. When combined with NVI grading, we were able to obtain sufficient neovascularization information.

Finally, our study with cadaveric eyes showed that IDCP led to histopathological structural changes only at the level of the ciliary epithelium, and no changes to the ciliary body muscle or deeper structures. This was consistent with the results of research by Pantcheva et al. [22], who performed cyclophotocoagulation with ECP (810 nm) on donor tissues. These findings indicated that both ECP (810 nm) and IDCP (532 nm) directly targeted the ciliary epithelium, and that termination based on the visible shrinkage and whitening of the target tissue minimized overtreatment. However, the response of donor eyes undergoing coagulation would be different from that produced in living eyes. This histopathological examination can only reflect the instantaneous changes after exposure to the laser.

The limitations of this study were its retrospective nature, the absence of a control group, the small sample size, midterm follow-up, and lack of the association between the photocoagulation ranges and the scope of IOP decrease. The long-term results are to be clarified; more research and a large, prospective, randomized, controlled clinical trial would further elucidate the appropriate use of combination treatments for managing NVG.

## 5. Conclusions

In this series of 25 eyes, the combined treatment including IDCP with 532 nm laser in patients with advanced NVG reduced IOP, medication use, and pain at all time points assessed. Our results show that IDCP using a 532 nm laser is a safe and effective way to perform internal cyclophotocoagulation without additional equipment.

## Figures and Tables

**Table 1:** Patient baseline characteristics.

Parameter	Total (*n* = 25)
No. of eyes/patients	25/24
Gender (F/M, no. of patients)	13/11
Age (yrs)	
Mean ± SD	58.3 ± 8.1
Range	42–70

Ischemic retinal disease, no. (%)	
PDR	16 (64)
CRVO	6 (24)
OIS	2 (8)
BCVA (LogMAR)	3.46 ± 0.81
IOP (mmHg), mean ± SD	42.2 ± 8.5

Previous treatment, no. (%)	
Complete PRP	3 (24)
Anti-VEGF	4 (16)
None	15 (60)
Topical antiglaucoma medication	2.72 ± 0.45

Lens status, no. (%)	
Phakia	22 (88)
Pseudophakia	3 (2)
Aphakia	0 (0)

NVI grade	
Grade 0	0 (0)
Grade 1	0 (0)
Grade 2	5 (20)
Grade 3	9 (36)
Grade 4	11 (44)

Anterior chamber angle	
180° closed	6 (24)
270° closed	8 (32)
360° closed	11 (44)

Values are presented as number, mean ± SD, or number (%). BCVA: best-corrected visual acuity; CRVO: central retinal vein occlusion; OIS: ocular ischemic syndrome; PDR: proliferative diabetic retinopathy; SD: standard deviation.

**Table 2:** Treatment results at 18-month follow-up.

Parameter	Total		*P* value
	Baseline	18 months	
No. of eyes	25	25	
BCVA (LogMAR)	3.46 ± 0.81	2.66 ± 1.05	<0.001
		3 (12%, stable)	
		22 (88%, improved)	

IOP (mmHg)	42.2 ± 8.5	15.56 ± 2.0	<0.001
Topical antiglaucoma medication	2.72 ± 0.45	0.87 ± 0.40	<0.001
NVI grade			
Grade 0	0 (0)	23 (92)	<0.001
Grade 1	0 (0)	2 (8)	
Grade 2	5 (20)	0 (0)	
Grade 3	9 (36)	0 (0)	
Grade 4	11 (44)	0 (0)	

Anterior chamber angle			
180° closed	6 (24)	6 (24)	
270° closed	8 (32)	8 (32)	
360° closed	11 (44)	11 (44)	

Values are presented as number (%) or mean ± SD. BCVA: best-corrected visual acuity; IOP: intraocular pressure; NVI: neovascularization of the iris. ^*∗*^*P* < 0.05 is considered statistically significant.

**Table 3:** Complications.

	*N* = 25
Anterior segment inflammation	14 (56%)
Hyphema	3 (12%)
Postoperative pain	0
Intraocular hemorrhage	0
Choroidal detachment	0
Hypotony	0
Total number of complications	17 (68%)

Hypotony was defined as intraocular pressure < 5 mmHg.

## Data Availability

The data used to support the findings of this study are available from the corresponding author upon request; please contact xminchen@whu.edu.cn for access.
